# Cytosolic nucleic acid sensors of the innate immune system promote liver regeneration after partial hepatectomy

**DOI:** 10.1038/s41598-018-29924-3

**Published:** 2018-08-16

**Authors:** Sarah Schulze, Christian Stöß, Miao Lu, Baocai Wang, Melanie Laschinger, Katja Steiger, Felicitas Altmayr, Helmut Friess, Daniel Hartmann, Bernhard Holzmann, Norbert Hüser

**Affiliations:** 1 Technical University of Munich, School of Medicine, Department of Surgery, Ismaninger Str. 22, 81675 Munich, Germany; 2 Technical University of Munich, School of Medicine, Comparative Experimental Pathology, Institute of Pathology, Trogerstr. 18, 81675 Munich, Germany

## Abstract

Stimulation of cytosolic nucleic acid sensors of innate immunity by pathogen-derived nucleic acids is important for antimicrobial defence, but stimulation through self-derived nucleic acids may contribute to autoinflammation and cancer. DNA sensing in the cytosol requires the stimulator of interferon genes (STING), while cytosolic RNA sensors use mitochondrial antiviral-signalling protein (MAVS). In a murine model of two-thirds hepatectomy, combined deficiency of MAVS and STING resulted in strongly impaired hepatocyte proliferation and delayed recovery of liver mass. Whereas lack of MAVS and STING did not influence upregulation of the G_1_-phase cyclins D1 and E1, it substantially reduced the hyperphosphorylation of retinoblastoma protein, attenuated the activation of cyclin-dependent kinase (CDK)-2, delayed upregulation of CDK1 and cyclins A2 and B1, and impaired S-phase entry of hepatocytes. Mechanistically, lack of cytosolic nucleic acid sensors strongly upregulated the anti-proliferative mediators TGF-β2 and activin A, which was associated with an increased expression of the cell cycle inhibitors p15 and p21. Partial hepatectomy was followed by the release of exosomes with abundant nucleic acid cargo, which may provide ligands for the MAVS and STING pathways. Together, these findings identify a previously unrecognised function of cytosolic nucleic acid sensors of innate immunity for promoting liver regeneration.

## Introduction

The innate immune system utilizes germline-encoded pattern recognition receptors to detect microbial threat and to initiate protective responses^1–3^. Innate pattern recognition receptors sense pathogens through detection of invariant microbial structures. In addition, pattern recognition receptors can be activated by host factors that are released from damaged tissues and, therefore, are regarded as danger-associated molecular patterns^4^. Signalling through innate pattern recognition receptors activates the production of inflammatory cytokines and type I interferons and induces the expression of costimulatory receptors on antigen-presenting cells. As a result, innate pattern recognition receptors orchestrate inflammatory responses and bridge innate and adaptive immune responses to combat microbial infection and to repair tissue injury.

Recognition of nucleic acids is considered important for an efficient innate immune response as nucleic acids may alert the innate immune system to the presence of living microbes or the uncontrolled death of host cells^5^. Innate immune sensors for nucleic acids are localized in either endosomes or the cytosol^3,6,7^. Endosomal nucleic acid sensors belong to the Toll-like receptor (TLR) family of pattern recognition receptors. While TLR3 acts as a sensor for double-stranded RNA, TLR7 and TLR8 recognise single-stranded RNA, and TLR9 detects bacterial and viral DNA. In the cytosol, retinoic acid-inducible gene-I-like receptors (RLRs) function as sensors of viral RNA and small endogenous RNAs. Signalling through RLRs is mediated by the adapter protein mitochondrial antiviral-signalling protein (MAVS), which is localized in the mitochondrial outer membrane. Protein interactions between RLRs and MAVS cause aggregation of MAVS and the subsequent activation of the transcription factors nuclear factor-κB (NF-κB) and interferon regulatory factor (IRF)-3.

Cytosolic DNA activates cyclic GMP-AMP synthase to synthesize the cyclic di-nucleotide cyclic GMP-AMP^6,8,9^. A single molecule of cyclic GMP-AMP binds to two molecules of the multispanning transmembrane protein stimulator of interferon genes (STING), which is anchored in the endoplasmic reticulum membrane at steady state. Upon binding of cyclic GMP-AMP, STING forms a complex with the kinase TBK1 and translocates to a perinuclear compartment. The translocation of STING requires autophagy-related genes and leads to the activation of TBK1 and IRF-3. Independently, cytosolic DNA may bind to the receptor absent in melanoma-2 (AIM2), leading to its interaction with the adaptor apoptosis-associated Speck-like protein and activation of the inflammasome.

Activation of cytosolic nucleic acid sensors was found to be important for the host defence against viral infection, but stimulation through accumulating self-nucleic acids may also contribute to various diseases, including autoinflammatory disorders and cancer^7,10–12^. The present study addresses the role of cytosolic nucleic acid sensing during liver regeneration. Using a standardized model of two-thirds partial hepatectomy, we have examined mice lacking both DNA and RNA sensing pathways due to a combined deficiency of the signalling adapter proteins MAVS and STING. The results demonstrate that deficiency of cytosolic nucleic acid sensing pathways markedly attenuated the recovery of liver mass following partial hepatectomy. Livers of mutant mice showed a delayed cell cycle progression. This was associated with an elevated expression of TGF-β2 and activin A, which, in turn was associated with an upregulation of the cell cycle inhibitors p15 and p21. Thus, cytosolic nucleic acid sensors promote liver regeneration after partial hepatectomy.

## Results

### Deficiency of MAVS and STING delays liver regeneration after partial hepatectomy

Nucleic acids signal the innate immune system the presence of living pathogens. However, self-nucleic acids released during tissue damage may represent danger-associated molecular patterns that contribute to autoinflammation and cancer. To examine the role of the cytosolic pathway of nucleic acid sensing for organ regeneration we established a mouse strain with a combined deficiency for the adapter proteins MAVS and STING (DKO mice). Consistent with previous studies targeting either MAVS^13–15^ or STING^16^ individually, genetic ablation of both adapter proteins was found to abrogate signalling downstream of cytosolic DNA as well as RNA sensors (Fig. S1).

**Figure 1:**
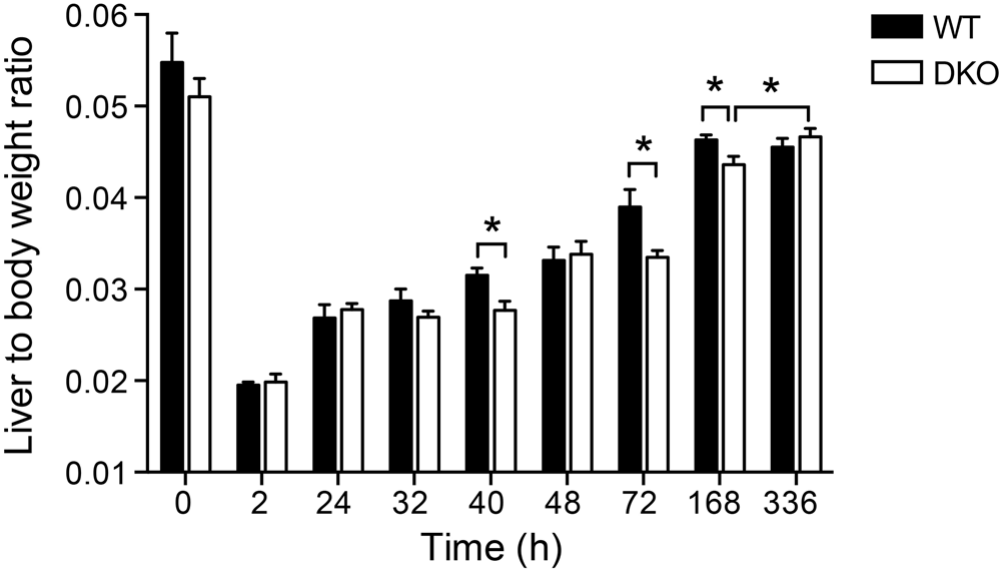
Lack of cytosolic nucleic acid sensing pathways impairs recovery of liver tissue after partial hepatectomy. Liver-to-body weight ratios were determined at the indicated time points following partial hepatectomy (n = 5 to 7 mice per time point in each group). *P < 0.05 (two-tailed unpaired Student’s t-test or the Mann-Whitney U test).

Without treatment, there were no differences detectable between wildtype and DKO livers with respect to liver morphology, including hepatocytes, biliary ducts, vessels or immune cell infiltration (Fig. S2). Moreover, untreated (0 h time point) wildtype and DKO mice showed similar liver-to-body weight ratios **(**Fig. 1**)**. When being examined in a model of two-thirds hepatectomy, we found that, also 2 h after hepatectomy, wildtype and DKO mice had similar liver-to-body weight ratios indicating a comparable loss of liver tissue **(**Fig. 1**)**. However, at later time points, liver regeneration was markedly impaired in DKO mice with liver-to-body weight ratios being significantly reduced at 40, 72 and 168 h after partial hepatectomy. Whereas wildtype mice reached plateau levels of liver-to-body weight ratios 168 h after partial hepatectomy with no further increase after 336 h, the relative liver mass of DKO mice significantly increased between 168 and 336 h. After 336 h, wildtype and DKO mice had similar liver-to-body weight ratios. These results show that cytosolic nucleic acid sensing mediated by the signalling adapters MAVS and STING is required for an efficient recovery of liver mass after partial hepatectomy.

### Lack of MAVS and STING enhances IL-6 production and early STAT3 signalling after partial hepatectomy

**Figure 2:**
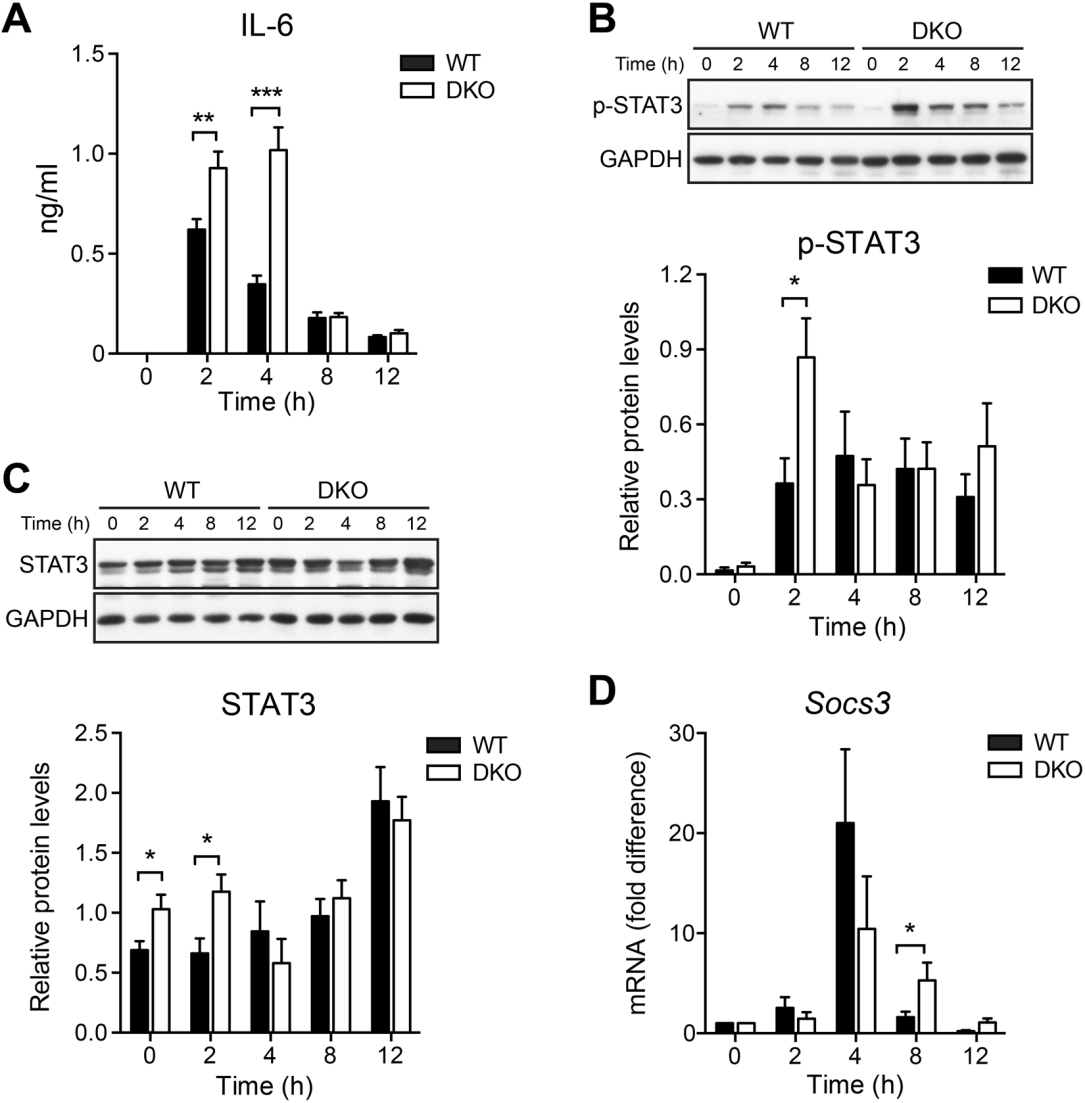
The IL-6 pathway and early STAT3 signalling are enhanced in the absence of cytosolic nucleic acid sensing. **(A)** Systemic levels of IL-6 were measured using serum samples from 4 to 7 independent mice for each time point and group. **(B)** Hepatic levels of phosphorylated STAT3 were determined by Western blotting. Representative gels (upper panel) and densitometric analyses (lower panel) are depicted. For each time point and in each group, samples from 6 independent mice were analysed. Cropped gels are shown. Full length gels are available in the supplemental information. **(C)** Expression of total STAT3 protein was determined by Western blotting. Representative gels (upper panel) and densitometric analyses (lower panel) are depicted. For each time point and in each group, samples from 6 independent mice were analysed. Cropped gels are shown. Full length gels are available in the supplemental information. **(D)** Expression of SOCS3 mRNA in livers following partial hepatectomy is depicted as fold difference relative to untreated livers. For each time point and in each group samples from 6 to 7 independent mice were analysed. *P < 0.05, **P < 0.01 ***P < 0.001 (two-tailed unpaired Student’s t-test or the Mann-Whitney U test).

IL-6 is produced during the first hours after partial hepatectomy and proposed to be important for the G_0_ to G_1_ cell cycle transition of hepatocytes^17–20^. We therefore examined whether cytosolic nucleic acid sensing may modulate liver regeneration by regulating IL-6 production. In wildtype mice, serum IL-6 levels showed a peak at 2 h after partial hepatectomy with a constant decline thereafter **(**Fig. 2A**)**. In DKO mice, IL-6 levels were significantly elevated both 2 and 4 h after partial hepatectomy, but did not differ from wildtype levels at other time points **(**Fig. 2A**)**.

The IL-6 receptor signals through the transducer and activator of transcription-3 (STAT3). Consistent with increased IL-6 levels, cellular levels of phosphorylated STAT3 were significantly enhanced in DKO mice at 2 h after partial hepatectomy **(**Fig. 2B**)**. In contrast, livers of untreated mice and post-hepatectomy livers derived from wildtype and DKO mice at later time points showed comparable levels of phosphorylated STAT3 **(**Fig. 2B**)**. In untreated controls and at 2 h after partial hepatectomy, but not at later time points, expression of total STAT3 protein was significantly elevated in DKO as compared with wildtype mice **(**Fig. 2C**)**. Expression of the STAT3 target gene suppressor of cytokine signalling-3 (SOCS3), which is known to negatively regulate STAT3, was significantly increased in DKO as compared with wildtype mice 8 h after partial hepatectomy **(**Fig. 2D**)**. Thus, the lack of cytosolic nucleic acid sensing leads to a transient increase in IL-6 production and signalling during the first few hours after partial hepatectomy. However, delayed liver regeneration in DKO cannot be explained by these effects.

### Hepatocyte proliferation is impaired in the absence of MAVS and STING

**Figure 3:**
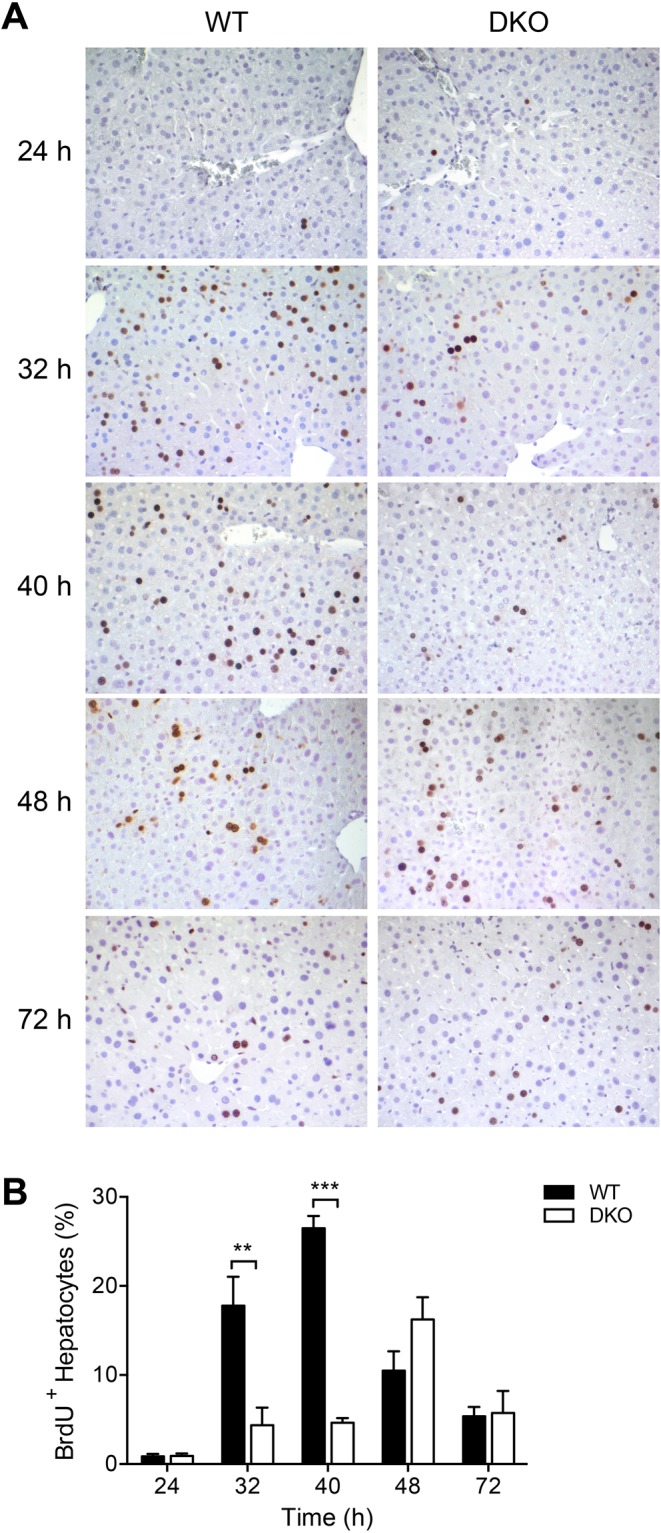
Entry of hepatocytes into S-phase of the cell cycle is impaired in mice with defective cytosolic nucleic acid sensing pathways. Representative immunohistochemical images **(A)** and quantification of the fraction of BrdU-positive hepatocytes **(B)** are shown. For each time point and in each group samples from 6 to 8 independent mice were analysed. **P < 0.01 ***P < 0.001 (two-tailed unpaired Student’s t-test or the Mann-Whitney U test).

Extensive proliferation of liver parenchymal cells is required to restore liver tissue after two-thirds hepatectomy^17,19–21^. Therefore, entry of hepatocytes into the S-phase of the cell cycle was examined by incorporation of 5-bromo-2′-deoxyuridine (BrdU). In WT mice, the proportion of BrdU-positive hepatocytes reached a maximum between 32 and 40 h after partial hepatectomy and decreased thereafter **(**Fig. 3A,B**)**. In DKO mice, however, kinetics of BrdU incorporation were markedly altered. As shown in Fig. 3A,B, the fraction of hepatocytes showing active DNA synthesis was significantly reduced in DKO as compared with wildtype mice both at 32 and 40 h after partial hepatectomy, and maximum BrdU incorporation in DKO livers was reached only after 48 h. Despite impaired S-phase entry in DKO hepatocytes, the overall tissue distribution of BrdU-positive hepatocytes was similar between WT and DKO mice **(**Fig. 3A**)**. Thus, the results indicate that entry of hepatocytes into the S-phase of the cell cycle following partial hepatectomy is both reduced and delayed in the absence of cytosolic nucleic acid sensing.

### Hepatocyte cell cycle progression is impaired in the absence of MAVS and STING

**Figure 4:**
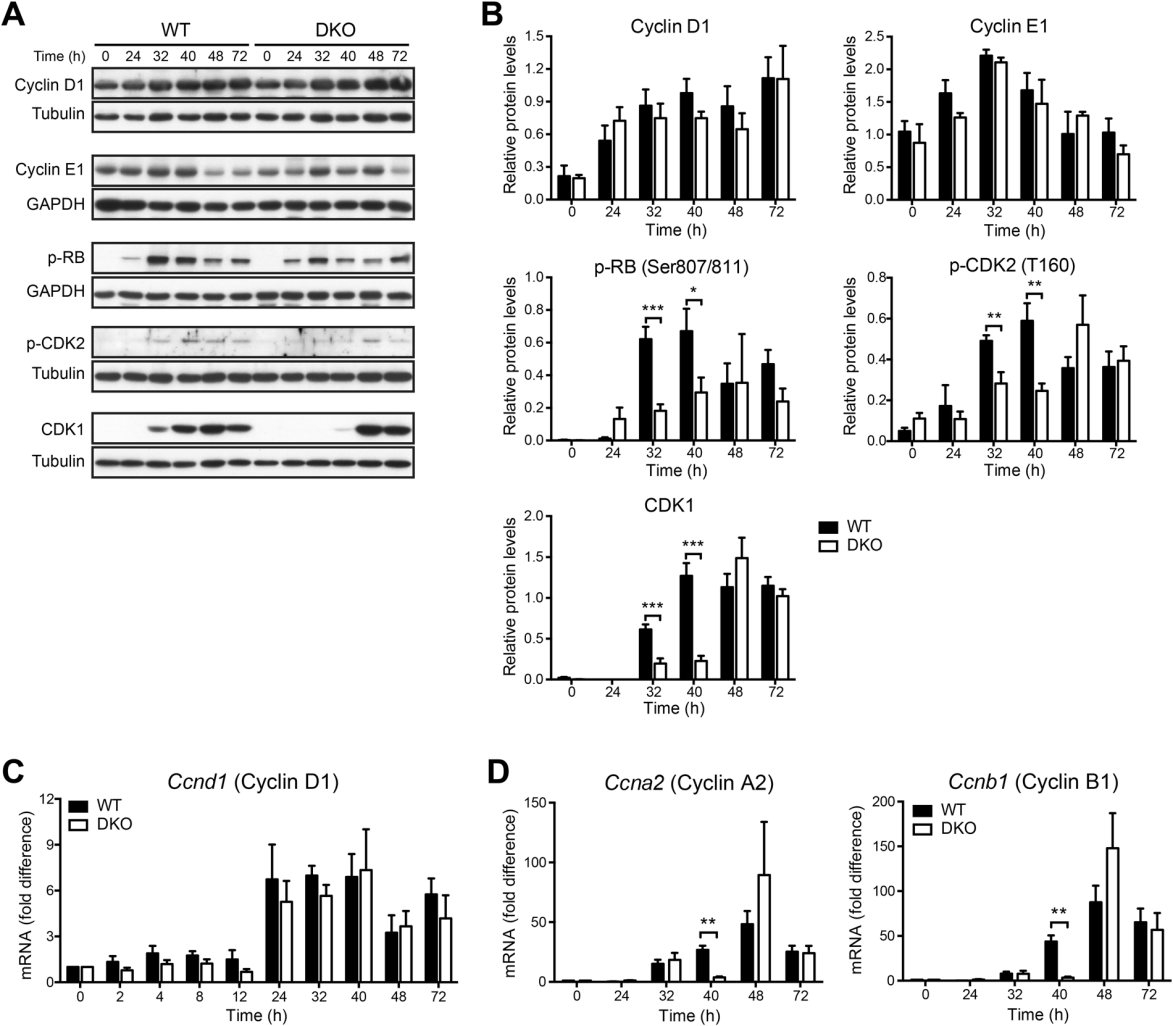
Impaired cell cycle progression in mice with defective cytosolic nucleic acid sensing pathways. Total protein extracts of liver samples were analysed by Western blotting using antibodies against cyclin D1, cyclin E1, p-RB, p-CDK2, or CDK1. Representative gels **(A)** and densitometric analyses **(B)** are depicted. Cropped gels are shown. Full length gels are available in the supplemental information. Hepatic mRNA expression of cyclin D1 **(C)** as well as cyclins A2 and B1 **(D)** is depicted as fold difference relative to untreated livers. For each time point and in each group samples from 4 to 8 independent mice were analysed. *P < 0.05, **P < 0.01 ***P < 0.001 (two-tailed unpaired Student’s t-test or the Mann-Whitney U test).

To elucidate potential mechanisms underlying the delayed hepatocyte cell cycle entry in DKO mice, we examined the expression of various cell cycle regulatory proteins. Cyclins D1 and E1 are upregulated during the early and late G_1_-phase of the cell cycle, respectively. As shown in Fig. 4A,B, cyclin D1 protein levels gradually increased from 24 to 72 h following partial hepatectomy and did not show significant differences between wildtype and DKO mice. Consistent with these findings, wildtype and DKO mice showed similar regulation of cyclin D1 mRNA levels at all time points analysed **(**Fig. 4C**)**. Cyclin E1 protein levels peaked at 32 h following partial hepatectomy and were also comparable between wildtype and DKO mice **(**Fig. 4A,B**)**. Thus, cytosolic nucleic acid sensing does not appear to influence the upregulation of G_1_ cyclins.

Inactivation of retinoblastoma (RB) protein through hyperphosphorylation is essential for cell cycle progression beyond the G_1_/S restriction point^22^. The results in Fig. 4A,B demonstrate that, in wildtype livers, phosphorylation of RB protein reached maximal levels at 32 h after partial hepatectomy and remained above unstimulated levels thereafter. In contrast, phosphorylation of RB protein was significantly impaired in livers of DKO mice both at 32 and 40 h after partial hepatectomy **(**Fig. 4A,B**)**. At later time points, RB phosphorylation was comparable between DKO and wildtype mice. These findings indicate that the combined deficiency of MAVS and STING results in a substantially decreased and delayed hyperphosphorylation of RB protein.

RB protein is phosphorylated and thereby inactivated by different G_1_ cyclin-dependent kinases, including cyclin-dependent kinase (CDK)-2 ref.^22^. Full activation of CDKs requires binding to cyclins as well as phosphorylation of a conserved threonine residue in the activation loop^23^. Consistent with RB protein phosphorylation, activating phosphorylation of CDK2 at Thr160 also increased at 32 and 40 h after partial hepatectomy in wildtype livers, but was significantly reduced in livers of DKO mice at these time points **(**Fig. 4A,B**)**. At later time points, wildtype and DKO mice showed comparable CDK2 phosphorylation levels. These findings show that the activating phosphorylation of CDK is markedly delayed in the absence of MAVS and STING.

To further analyse the role of MAVS and STING in cell cycle progression, expression of late stage cyclins (A and B) and CDK1 was determined. The results in Fig. 4D demonstrate that induction of cyclin A2 and B1 mRNA levels was delayed in DKO mice with significantly lower expression at the 40 h time point as compared with wildtype mice. Moreover, upregulation of CDK1 was also severely delayed in DKO mice. In DKO mice, CDK1 protein expression was significantly reduced at 32 and 40 h, but not at later time points as compared with wildtype controls **(**Fig. 4A,B**)**. Together, these results indicate that, following partial hepatectomy, cytosolic nucleic acid sensing mediated by the signalling adapter proteins MAVS and STING is required for a normal cell cycle progression of hepatocytes.

### Deficiency of MAVS and STING causes enhanced expression of cell cycle inhibitors

**Figure 5:**
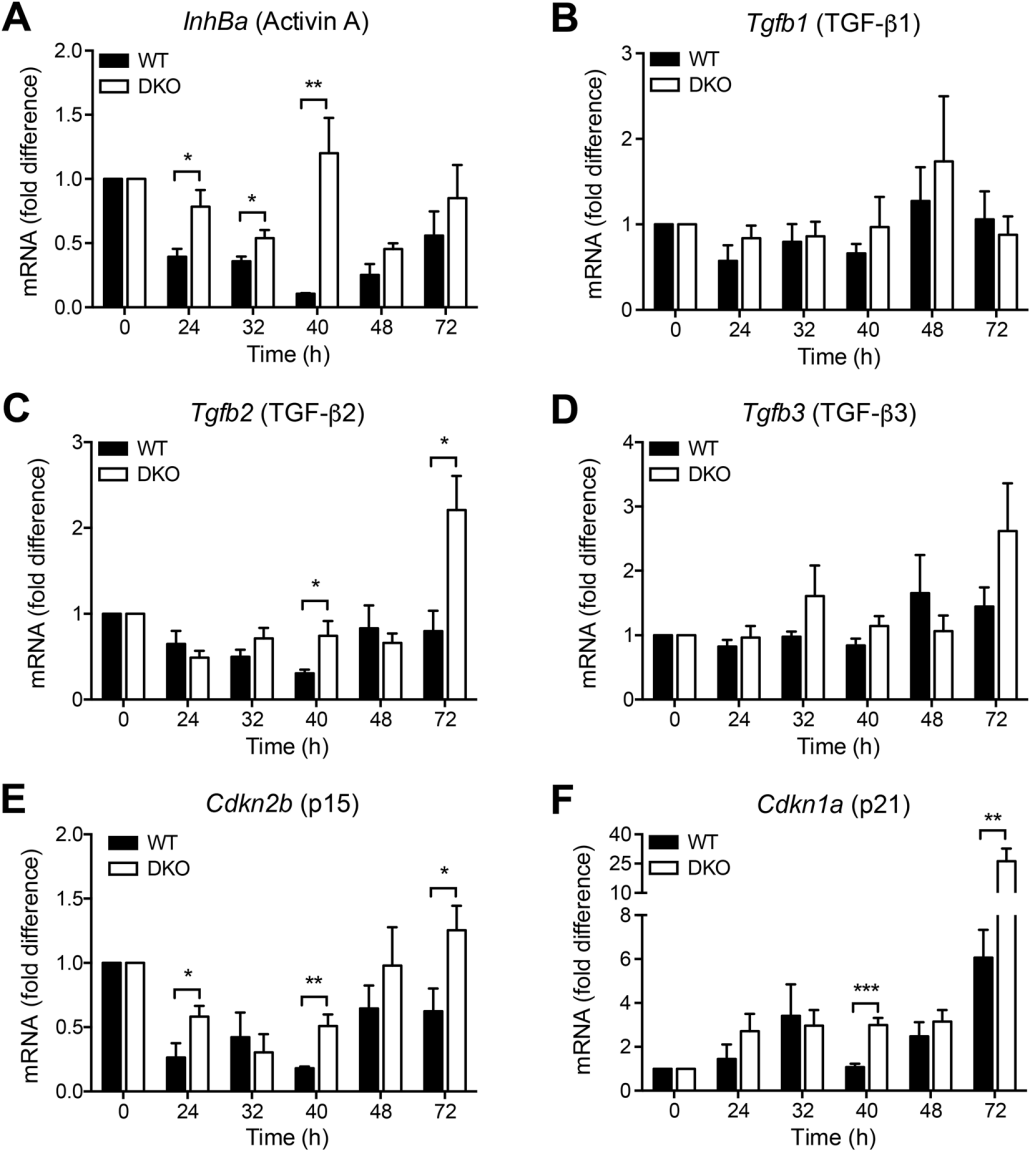
Lack of cytosolic nucleic acid sensing enhances expression of cell cycle inhibitors. Hepatic mRNA expression of activin A **(A)**, TGF-β1 **(B)**, TGF-β2 **(C)**, TGF-β3 **(D)**, p15 **(E)**, and p21 **(F)** at various time points after partial hepatectomy is depicted as fold difference relative to untreated livers. For each time point and in each group samples from 5 to 7 independent mice were analysed. *P < 0.05, **P < 0.01 ***P < 0.001 (two-tailed unpaired Student’s t-test or the Mann-Whitney U test).

Previous work has shown that hepatocyte proliferation during liver regeneration may be inhibited by TGF-β and activin A^24–27^. We have therefore examined whether cytosolic nucleic acid sensing involving MAVS and STING may influence expression of these mediators. In wildtype mice, we observed a massive downregulation of activin A mRNA levels with lowest expression at 40 h after hepatectomy followed by a continuous increase at 48 and 72 h **(**Fig. 5A**)**. Importantly, downregulation of activin A mRNA was severely impaired in DKO mice resulting in a significantly increased expression at 24, 32, and 40 h, but not at later time points **(**Fig. 5A**)**. The TGF-β1 and TGF-β3 isoforms showed comparable expression in wildtype and DKO mice **(**Fig. 5B,D**)**. In contrast, the time course of TGF-β2 mRNA expression was significantly elevated both at 40 and 72 h after partial hepatectomy in DKO mice as compared with wildtype mice **(**Fig. 5C**)**. Thus, these results indicate that the absence of MAVS and STING results in an increased expression of activin A and TGF-β2 during the course of liver regeneration.

**Figure 6:**
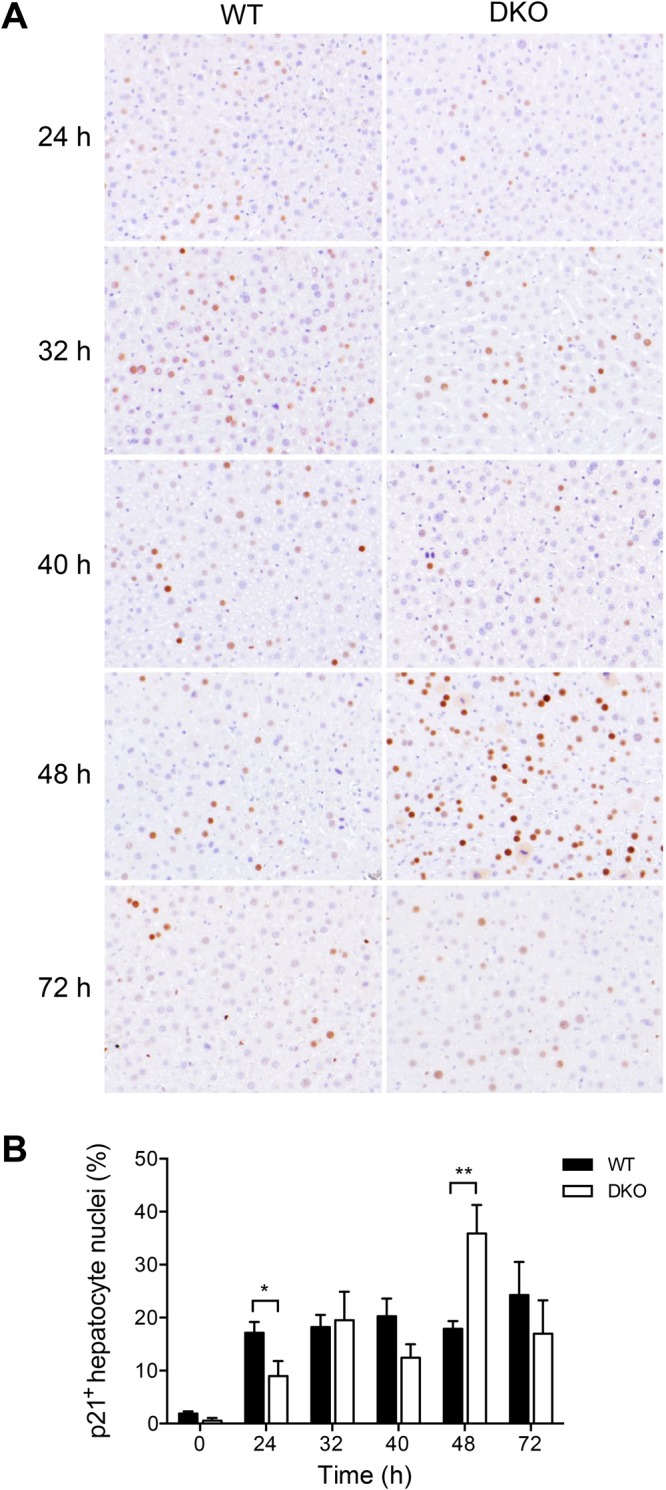
Influence of cytosolic nucleic acid sensing pathways on p21 protein expression during liver regeneration. Representative immunohistochemical images **(A)** and quantification of the fraction of hepatocytes showing nuclear p21 expression **(B)** are shown. For each time point and in each group samples from 6 independent mice were analysed. *P < 0.05, **P < 0.01 (two-tailed unpaired Student’s t-test or the Mann-Whitney U test).

TGF-β family members including activin A are known to induce expression of the small cell cycle inhibitors p15 and p21 ref.^28^. The results in Fig. 5E demonstrate that, in wildtype mice, p15 mRNA was downregulated after partial hepatectomy. Importantly, p15 mRNA levels were significantly increased in livers of DKO mice as compared with wildtype mice at the 24, 40, and 72 h time points **(**Fig. 5E**)**. Expression of p21 showed a biphasic regulation in wildtype mice with a first increase at 32 h and a late peak at 72 h **(**Fig. 5F**)**. Similar to p15 expression, p21 mRNA levels were significantly increased in livers of DKO mice as compared with wildtype controls both 40 and 72 h after partial hepatectomy **(**Fig. 5F**)**. To corroborate these findings on the protein level, expression of p21 was investigated by immunohistochemistry. The results in Fig. 6A,B show that, similar to total organ mRNA levels, nuclear expression of p21 in hepatocytes was increased during liver regeneration. Expression of p21 was weak at 24 h after partial hepatectomy, but was elevated in wildtype as compared with DKO mice. Hepatocyte nuclear p21 expression in DKO mice reached wildtype levels at 32 and 40 h. At 48 h after partial hepatectomy, however, the fraction of p21-positive hepatocyte nuclei was significantly increased in DKO as compared with wildtype mice. Considered together, these results indicate that the signalling adapter proteins MAVS and STING are essential for attenuating the expression of the anti-proliferative mediators TGF-β2 and activin A, which, in turn, may control the small cell cycle inhibitors p15 and p21.

### Extracellular vesicles are released during liver regeneration

**Figure 7:**
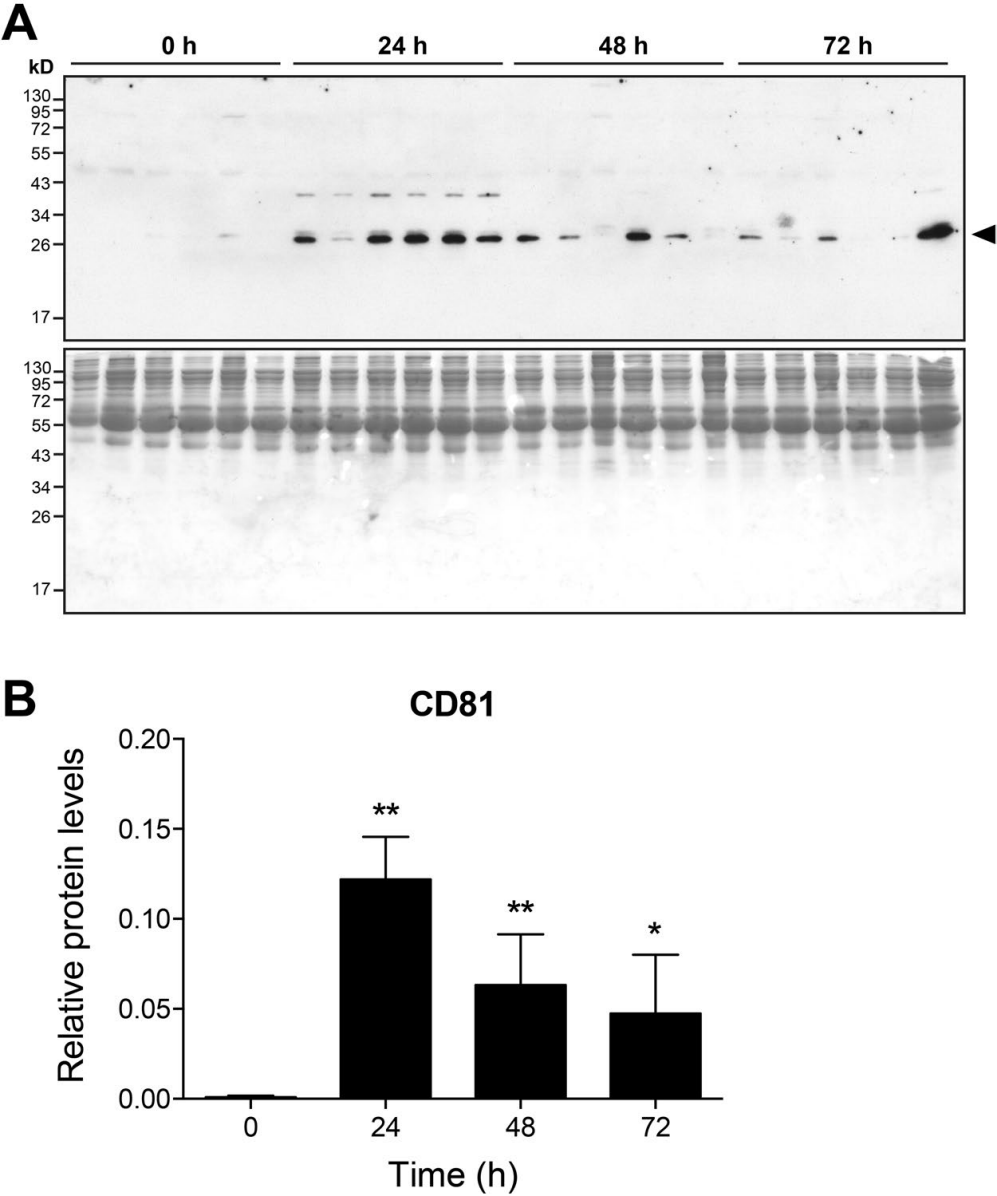
Release of CD81-positive extracellular vesicles during liver regeneration. Exosomes were isolated from serum samples using ExoQuick and exosome lysates were analysed by Western blotting using an antibody against CD81 (A, upper panel). The protein band corresponding to CD81 is indicated by an arrowhead. Protein loading was controlled by coomassie blue staining of gels (A, lower panel). For each time point, samples from six individual wildtype mice are shown. Full length gels are depicted. Expression of CD81 was quantified by densitometric analyses (**B**). *P < 0.05, **P < 0.01 (two-tailed unpaired Student’s t-test or the Mann-Whitney U test).

Administration of exosomes derived from *in vitro* cultured hepatocytes to mice was shown to enhance liver regeneration upon partial hepatectomy^29^. Numerous studies have demonstrated that extracellular vesicles such as exosomes are important in liver pathology and contain self nucleic acids that may stimulate cytosolic nucleic acid sensors after vesicle fusion with target cells^30–32^. Thus, extracellular vesicles released during liver regeneration may serve as a potential source of immunostimulatory nucleic acids activating MAVS- and STING-dependent signalling pathways. To address this hypothesis exosomes were enriched from serum samples of wildtype mice at various time points after partial hepatectomy. Lysates of exosome preparations were subjected to Western blot analysis using an antibody against the tetraspanin CD81, which is a known exosomal protein and is strongly expressed in hepatocyte-derived exosomes^33^. The results in Fig. 7A,B show that exosomal CD81 is barely detectable in sera of untreated mice, but is abundant 24 h after partial hepatectomy. At the 48 and 72 h time points, CD81 expression declined, but remained significantly increased **(**Fig. 7B**)**. In addition, we intended to confirm that exosomes may serve as nucleic acid carriers during liver regeneration. To this end, total nucleic acids were isolated from exosomes derived from serum samples of wildtype mice 24 h after partial hepatectomy, because exosomes were found to be most abundant at this time point **(**Fig. 7**)**. Our results directly demonstrated that exosomes isolated from 200 µl of serum contained 4.592 ± 0.763 µg total nucleic acids (mean ± standard error of the mean, n = 5). Together, these results indicate that liver regeneration after partial hepatectomy is associated with an early and intense release of exosomes containing an abundant nucleic acid cargo, which may stimulate MAVS- and STING-dependent nucleic acid sensors.

## Discussion

Cytosolic nucleic acid sensors of the innate immune system are considered important for antimicrobial defence, but their activation through endogenously derived nucleic acids may contribute to autoinflammatory disorders and cancer^7,10–12^. Supporting the role of endogenous nucleic acids as danger-associated molecular patterns, genetic deficiencies of nucleases or RNA-editing enzymes in human patients result in an accumulation of endogenous immunostimulatory nucleic acids, leading to the Aicardi-Goutieres syndrome or some forms of Systemic lupus erythematosus. Moreover, activating mutations of STING may cause severe inflammatory vasculopathies with onset in infancy. In mice, deletions of DNA nucleases lead to inflammatory disorders, which are dependent on STING expression. Analysis of murine cancer models further suggests that STING promotes chemical carcinogen-induced skin polyp formation, but exerts protective effects in models of colitis-associated carcinogenesis. In the present study, we provide direct evidence that cytosolic nucleic acid sensors of the innate immune system have an important and previously unrecognised role in organ regeneration. Analysis of mice with a combined deficiency of the adapter proteins MAVS and STING in a standardized model of two-thirds hepatectomy demonstrates that cytosolic nucleic acid sensing pathways are required for normal hepatocyte cell cycle progression and efficient recovery of organ mass during liver regeneration.

Although cytosolic nucleic acid sensors are required for liver regeneration after partial hepatectomy, we found that, in the resting state, DKO mice do not appear to have a liver phenotype. Thus, liver-to-body weight ratios, liver histology, and hepatic protein levels of cyclin D1, cyclin E1, CDK1, p-CDK2 and p-RB were not significantly different between DKO and wildtype mice. In contrast, total STAT3 protein levels were weakly increased in DKO as compared with wildtype mice in the resting state. However, it should be noted that serum IL-6 and hepatic p-STAT3 levels in untreated DKO mice were normal indicating that elevated STAT3 expression does no lead to an enhanced basal signalling activity.

Liver regeneration after partial hepatectomy requires an extensive and coordinated proliferation of hepatocytes. Analysis of BrdU incorporation revealed that the lack of MAVS and STING substantially delayed the entry of hepatocytes into the S-phase of the cell cycle, thereby providing an explanation for the delayed recovery of organ mass in DKO mice. Consistent with these findings, upregulation of cyclin A2, cyclin B1, and CDK1, which control S-phase progression and/or the transition from the S-to the M-phase of the cell cycle, was also significantly attenuated. In contrast, protein expression of cyclins D1 and E1, which are induced during the G_1_-phase of the cell cycle, was not altered in DKO mice. These results suggest that DKO hepatocytes enter the G_1_-phase but are impaired in their capacity to progress to the S-phase. Hyperphosphorylation of RB protein by G_1_ cyclin/CDK complexes is important for G_1_-to S-phase transition of the cell cycle^22^. It was therefore important to discover that both hyperphosphorylation of RB protein and the activating phosphorylation of CDK2 at Thr160 were strongly reduced in livers of DKO mice. Together, these findings indicate that impaired hepatocyte cell cycle progression after partial hepatectomy in MAVS- and STING-deficient mice is linked to defects in the activation of the cyclin E-CDK2 complex and an inactivation of RB protein.

The three TGF-β isoforms were found to be expressed in regenerating rat livers with expression being detected exclusively in nonparenchymal cells^34^. Direct evidence for a role of TGF-β in liver regeneration was provided by analysis of mice deficient for the TGF-β receptor II, which exhibited increased hepatocyte proliferation and accelerated recovery of liver mass after partial hepatectomy^24,26^. Whereas in one study defective TGF-β signalling was also shown to impair termination of liver regeneration^26^, another study reported that this activity was compensated by an increased expression of activin A and its type II receptor^24^. It was therefore interesting to find out in our studies that, in livers of DKO mice, expression of activin A was persistently elevated between 24 and 40 h after hepatectomy and that TGF-β2 levels were increased at the 40 and 72 h time points. Thus, our findings identify cytosolic nucleic acid sensing pathways as novel regulators of TGF-β2 and activin A during liver regeneration and suggest that delayed liver regeneration in DKO mice is associated with an impaired control of these anti-proliferative mediators.

TGF-β isoforms as well as activin A are known to induce expression of the cell cycle inhibitors p15 and p21 ref.^28^. Importantly, and correlating with the expression of TGF-β2 and activin A, mRNA expression of both p15 and p21 was strongly upregulated in DKO as compared with wildtype mice at various time points after partial hepatectomy. Moreover, hepatocytes in regenerating livers of DKO showed an elevated nuclear expression of p21 protein. Previous work revealed that the cell cycle inhibitor p21 is upregulated during liver regeneration and that genetic ablation of p21 accelerates the recovery of liver tissue after partial hepatectomy^35^. Considered together, these results therefore suggest that defective nucleic acid sensing impairs liver regeneration through increasing expression of the anti-proliferative mediators TGF-β2 and activin A, which, in turn, is associated with an upregulation of the cell cycle inhibitors p15 and p21.

Innate pattern recognition receptors of the TLR family that signal through the adapter protein MyD88 were shown to influence liver regeneration by regulating IL-6 production during the priming phase after partial hepatectomy^36–38^. Early induction of IL-6 is considered important, because liver regeneration is severely impaired in mice lacking IL-6^39^. The results of the present study demonstrate that, unlike the MyD88 pathway, the lack of MAVS and STING signalling transiently enhanced IL-6 production. A possible explanation is provided by a recent study showing that the transcription factor IRF-3, which is activated downstream of MAVS and STING, may prevent activation of NF-κB by blocking the kinase domain of IKKβ^40^. However, enhanced IL-6 production in the absence of MAVS and STING would be expected to promote rather than impair liver regeneration and, therefore, does not explain the observed phenotype of DKO mice. Instead, enhanced IL-6 production may help to counteract, at least to a limited extent, the inhibitory effects of combined MAVS and STING deficiency on liver regeneration.

Similar to signalling triggered by MAVS and STING, ligand binding to TLR3 may also lead to the activation of IRF-3 and the production of type I interferons^41,42^. It is therefore interesting to note that deficiency of TLR3 accelerates rather than delays hepatocyte proliferation after partial hepatectomy^43^. However, it should be considered that TLR3 signalling may, in addition to IRF-3 stimulation, cause activation of PI3 kinase^44^, AKT^45^, and caspase 8 ref.^46,47^ and contributes to epigenetic regulation of gene expression^48^. Moreover, TLR3 and cytosolic nucleic acid sensors exhibit, at least in part, a distinct cellular distribution in liver. TLR3 shows a broad expression, which includes hepatocytes, biliary epithelial cells, sinusoidal endothelial cells and Kupffer cells^49^. Whereas MAVS is also expressed by hepatocytes^13^, STING was reported to be absent^50^. Both MAVS and STING are found in Kupffer cells, but expression in other non-parenchymal cells has not been described. Thus, it is conceivable that different functions of TLR3 and MAVS or STING in liver regeneration are also related to differences in cell types involved, although it should be noted that cell type-specific contributions of these proteins have yet to be determined.

Self nucleic acids enter cells for recognition by innate immune sensors after forming complexes with antimicrobial peptides or antibodies, or through the uptake of extracellular vesicles^32^. In addition, cellular stress may cause cytosolic accumulation of mitochondrial and nuclear DNA. In the liver, extracellular vesicles are released under various physiologic and pathologic conditions and exert a wide range of effects^30,31^. Specifically, microRNAs that are associated with the vesicular fraction of the serum are increased in the blood circulation during the proliferation phase of liver regeneration after partial hepatectomy and administration of exosomes derived from cultured hepatocytes to hepatectomized mice may enhance liver regeneration^29,51^. Intriguingly, a recent study has shown that exosomes released from tumour stromal cells contain unshielded RNA that, upon exosome transfer to breast cancer cells, activates the retinoic acid-inducible gene-I/MAVS pathway to enhance tumour growth and metastasis^52^. We therefore hypothesized that extracellular vesicles released during liver regeneration deliver immunostimulatory nucleic acids to target cells, which causes activation of cytosolic nucleic acid sensors and results in an improved liver regeneration. Our finding that exosomal vesicles are highly abundant during the proliferation phase of liver regeneration and contain considerable amounts of nucleic acids are consistent with this hypothesis.

## Methods

### Animals

*Mavs*^−/−^13 and *Tmem173*^gt^ ref.^53^ single mutant mice were maintained on a C57BL/6N background. Mice with a combined homozygous deficiency of the signalling adapter proteins MAVS and STING were generated by intercross of *Mavs*^-/-^ and *Tmem173*^gt^ mice. Mouse colonies were housed in a specified pathogen-free facility (Charles River Laboratories SRL, Calco, Italy). C57BL/6N wildtype control mice were obtained from Charles River. Male mice at the age of 10–12 weeks kept on a 12 h day/night cycle with free access to food and water were used in all experiments.

### Partial hepatectomy

Mice were subjected to two-thirds partial hepatectomy using standard procedures^54^. According to this protocol, ligation and resection of the median lobe and the left lateral lobe were performed separately. Mice were anesthetized under isofluorane inhalation. Hepatectomies were performed between 8 and 10 am. At the indicated time points, mice were sacrificed and remnant livers were collected for further analyses. Animal experiments were performed in accordance with Federal Animal Regulations and were institutionally approved by the District Government of Upper Bavaria (AZ 55.2.1.54-2532-147-2015).

### Western blotting

Liver samples were homogenized in tissue lysis buffer using a TissueLyser II instrument (Qiagen, Hilden, Germany). The tissue lysis buffer contained 1% Triton X-100, 150 mM NaCl and 20 mM Tris-HCl pH 7.5 and was supplemented with protease and phosphatase inhibitors. Lysates were separated by SDS-PAGE and transferred to a nitrocellulose membrane. Membranes were incubated with the following primary antibodies: STAT3, p-STAT3 (Tyr705), p-RB (S807/811), p-CDK2 (T160), cyclin E1, and GAPDH (all from Cell Signaling Technology, Danvers, MA); β-tubulin and CDK1 (both from Abcam, Cambridge, MA); CD81 (St. John’s Laboratory, London, UK); and cyclin D1 (Santa Cruz Biotechnology, Dallas, TX). Second stage goat-anti-rabbit-HRP was obtained from Jackson ImmunoResearch Laboratories Inc. Antibody binding was visualized using the Pierce™ ECL western blotting detection system (Thermo Fisher Scientific, Waltham, MA). Densitometric analyses were performed using the ImageJ software (https://imagej.nih.gov/ij/).

### Quantitative reverse transcriptase PCR

Liver samples were snap frozen in liquid nitrogen immediately after explantation and stored at −80 °C until use. RNA was prepared using the RNeasy Mini Kit (Qiagen, Hilden, Germany). First-strand cDNA was synthesized from 1 mg total RNA using the QuantiTect Reverse Transcription Kit (Qiagen, Hilden, Germany). Quantitative reverse transcriptase-PCR analyses were performed using the Universal Probe Library (Roche Diagnostics, Indianapolis, IN). The primers were: *Cdkn1a* sense, 5′-AAC ATC TCA GGG CCG AAA-3′; *Cdkn1a* antisense, 5′-TGC GCT TGG AGT GAT AGA AA-3′; *Socs3* sense, 5′-ATT TCG CTT CGG GAC TAG C-3′; *Socs3* antisense, 5′-AAC TTG CTG TGG GTG ACC AT-3′; *Cdkn2b* sense, 5′-AAT AAC TTC CTA CGC ATT TTC TGC-3′; *Cdkn2b* antisense, 5′-CCC TTG GCT TCA AGG TGA G-3′; *Ccnd1* sense, 5′-TCT TTC CAG AGT CAT CAA GTG TG-3′; *Ccnd1* antisense, 5′-GAC TCC AGA AGG GCT TCA ATC-3′; *Ccna2* sense, 5′-CTT GGC TGC ACC AAC AGT AA-3′; *Ccna2* antisense, 5′-CAA ACT CAG TTC TCC CAA AAA CA-3′; *Ccnb1* sense, 5′-GCT TAG CGC TGA AAA TTC TTG-3′; *Ccnb1* antisense, 5′-TCT TAG CCA GGT GCT GCA TA-3′; *Tgfb1* sense, 5′-CCT TCC TGC TCC TCA TGG-3′; *Tgfb1* antisense, 5′-CGC ACA CAG CAG TTC TTC TC-3′; *Tgfb2* sense, 5′-AGG AGG TTT ATA AAA TCG ACA TGC-3′; *Tgfb2* antisense, 5′-TAG AAA GTG GGC GGG ATG-3′; *Tgfb3* sense, 5′-CCC TGG ACA CCA ATT ACT GC-3′; *Tgfb3* antisense, 5′-TCA ATA TAA AGG GGG CGT ACA-3′; *InhBa* sense, 5′-ATC ATC ACC TTT GCC GAG TC-3′; *InhBa* antisense, 5′-TCA CTG CCT TCC TTG GAA AT-3′; *Actb* sense, 5′-AAG GCC AAC CGT GAA AAG AT-3′; *Actb* antisense, 5′-GTG GTA CGA CCA GAG GCA TAC-3′. RNA levels were normalized to those of *Actb* (β-actin) and are depicted as fold difference relative to liver samples of untreated mice. Accumulation of PCR amplicons was quantified on a LightCycler 480 Real-Time PCR system (Roche Diagnostics, Indianapolis, IN).

### BrdU labelling and immunohistochemistry

Mice were given intraperitoneal injections of 100 µg/g 5-bromo-2′-deoxyuridine (BrdU; Roche Diagnostics, Indianapolis, IN) 2 h before sacrifice. Liver samples were fixed for 24 h in 4% paraformaldehyde, dehydrated in a graded alcohol series and embedded in paraffin. Sections were incubated with anti-BrdU monoclonal antibody (Merck Millipore, Billerica, MA) or a rabbit monoclonal antibody against p21 (Abcam, Cambridge, MA) and stained using the Dako EnVision^+^ System (Agilent Technologies, Santa Clara, CA). For each animal, five to six random high power fields were counted and the fraction of stained hepatocyte nuclei was calculated.

### IL-6 serum levels

IL-6 protein concentrations in serum were determined using the Quantikine® ELISA Mouse IL-6 Immunoassay (R&D Systems, Minneapolis, USA) according to manufacturer’s instructions.

### Exosome preparation

Exosomes were isolated from serum samples using ExoQuick according to the instructions of the manufacturer (System Biosciences, Mountain View, CA). Briefly, 100 µl of serum were mixed with 25 µl of ExoQuick and incubated at 4 °C for 30 minutes. Samples were centrifuged at 13.000 rpm for 2 minutes and exosome pellets were resuspended in ExoQuick lysis buffer. For western blot analysis, 40 µg protein of each sample were separated by SDS-PAGE. From exosome preparations of independent serum samples, total nucleic nucleic acids were isolated using the Trizol method. Nucleic acid concentrations were determined photometrically.

### Statistical analysis

All data are presented as means ± standard error of the mean. Statistical differences were analysed using the two-tailed unpaired Student’s t-test or the Mann-Whitney U test. Differences between experimental groups were considered significant, when the *P* values were < 0.05.

### Data availability

All data generated or analysed during this study are included in this published article (and its Supplementary Information files).

## Electronic supplementary material

Supplementary information
