# Supplementary material for: Cytosolic nucleic acid sensors of the innate immune system promote liver regeneration after partial hepatectomy
Source: Sci Rep. 2018 Aug 16;8:12271. doi: 10.1038/s41598-018-29924-3 (PMC6095902; doi:10.1038/s41598-018-29924-3)
Supplement: Supplementary file 1 — Supplementary information [file 41598_2018_29924_MOESM1_ESM.pdf]

Cytosolic nucleic acid sensors of the innate immune system promote liver regeneration after partial hepatectomy

Sarah Schulze [1], Christian Stöß [1], Miao Lu [1], Baocai Wang [1], Melanie Laschinger [1], Katja Steiger [2], Felicitas Altmayr [1], Helmut Friess [1], Daniel Hartmann [1], Bernhard Holzmann [1 + *], Norbert Hüser [1 +]

[1] Technical University of Munich, School of Medicine, Department of Surgery, Ismaninger Str. 22, 81675 Munich, Germany

[2] Technical University of Munich, School of Medicine, Comparative Experimental Pathology, Institute of Pathology, Trogerstr. 18, 81675 Munich, Germany

* Corresponding author: bernhard.holzmann@tum.de

[+] These authors contributed equally to this study

Supplemental Figure S1

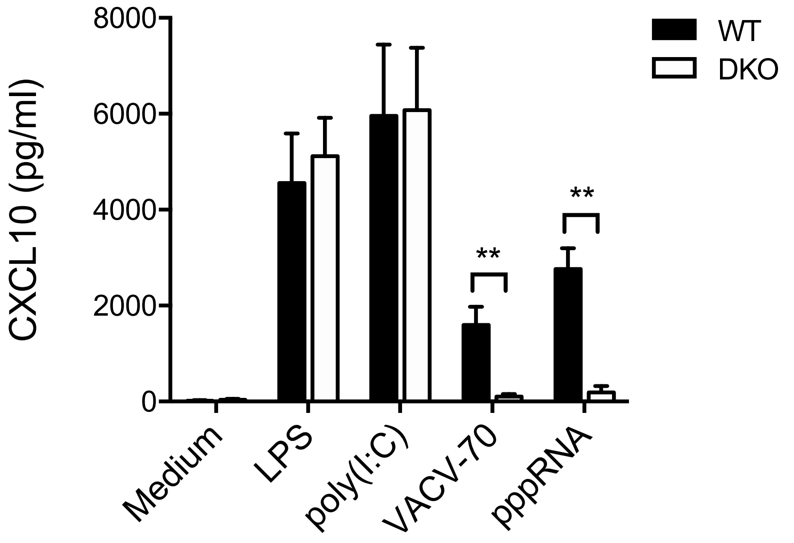

Legend:

Bone marrow-derived macrophages from wildtype and DKO mice were generated by culture of bone marrow cells in RPMI1640 medium containing 10 % fetal calf serum and 15 % L cell-conditioned medium as a source of M-CSF for at least 7 d.

For controls, cells were used unstimulated (medium) or were stimulated with 10 ng/ml of the TLR4 agonist *E.coli* LPS (Sigma-Aldrich Chemie GmbH, Taufkirchen, Germany) or 50 µg/ml of the TLR3 agonist polyI:C (Invivogen, Toulouse, France).

To stimulate cytosolic DNA sensors, macrophages were incubated with 3 µg/ml of the immunostimulatory DNA VACV-70 that was complexed with the transfection reagent LyoVec™ to facilitate its uptake (Invivogen, Toulouse, France).

To stimulate cytosolic RNA sensors, macrophages were incubated with 1 µg/ml 5'ppp-dsRNA that was complexed with the transfection reagent LyoVec™ to facilitate its uptake (Invivogen, Toulouse, France).

After 16 h of stimulation, CXCL10 levels were measured in culture supernatants using the Mouse CXCL10 Quantikine ELISA Kit (R&D Systems, Minneapolis, USA) according to manufacturer's instructions.

Supplemental Figure S2

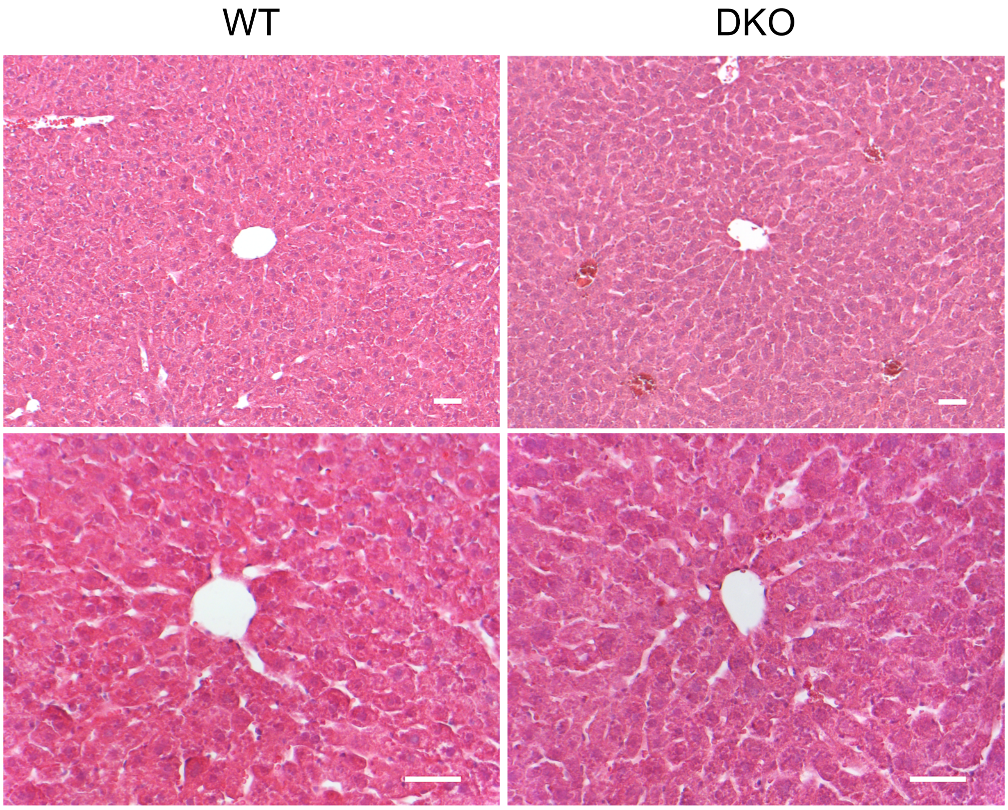

Legend:

Liver sections of untreated wildtype and DKO mice (n=6) were stained with hematoxylin/eosin and mounted with Eukitt mounting medium (Merck, Darmstadt, Germany). Stained sections were imaged using an Axiolap microscope attached to an AxioCam MRc5 camera, an EC Plan-Neofluar 10x/0.3 NA and an Achroplan 20x/0.45 NA objective and analyzed using the Axiovision software (all Carl Zeiss Microscopy, Jena, Germany).

Representative images of wildtype and DKO mice are depicted. Scale bars indicate 50µm.

Full length gels for Figure 2B

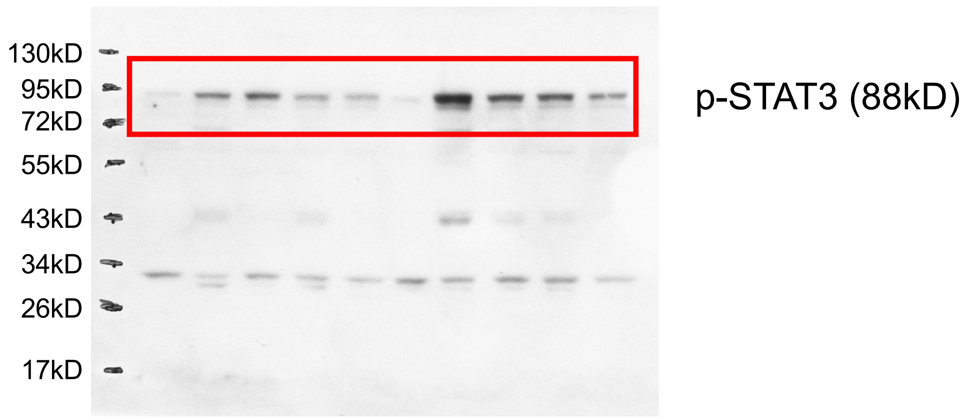

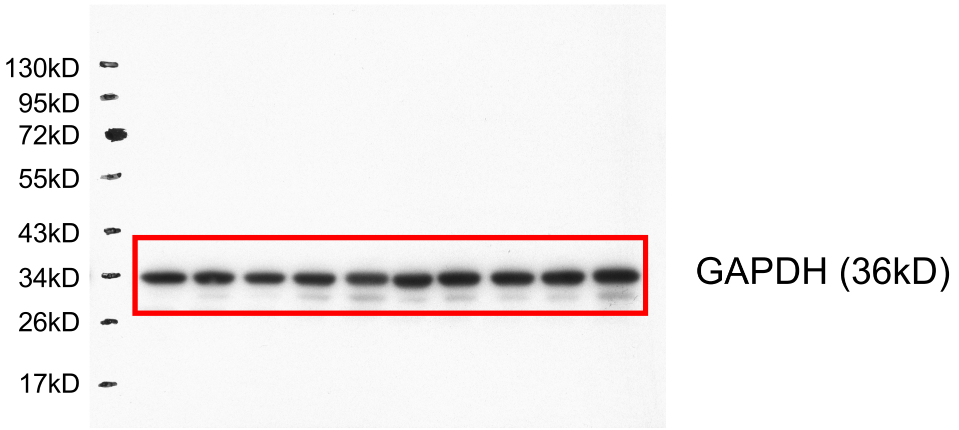

Legend:

Areas of the gel shown in Figure 2B are marked in red.

The membrane was probed first with anti-p-STAT3 and subsequently with anti-GAPDH antibodies. Predicted molecular weights for p-STAT3 and GAPDH are indicated.

Full length gels for Figure 2C

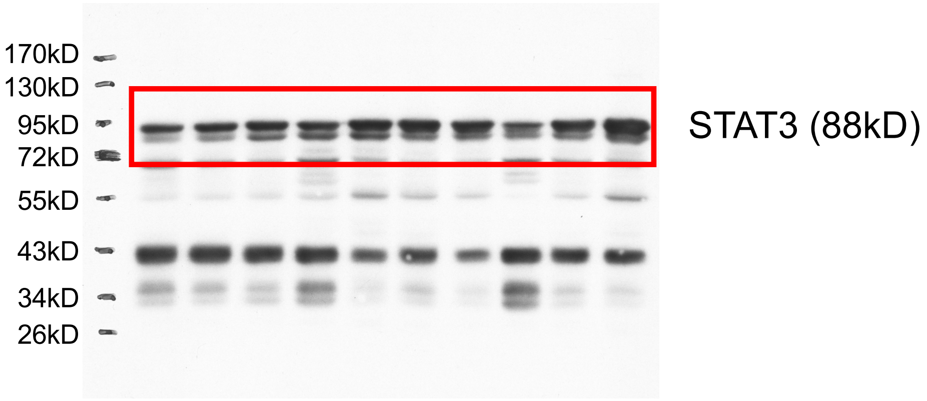

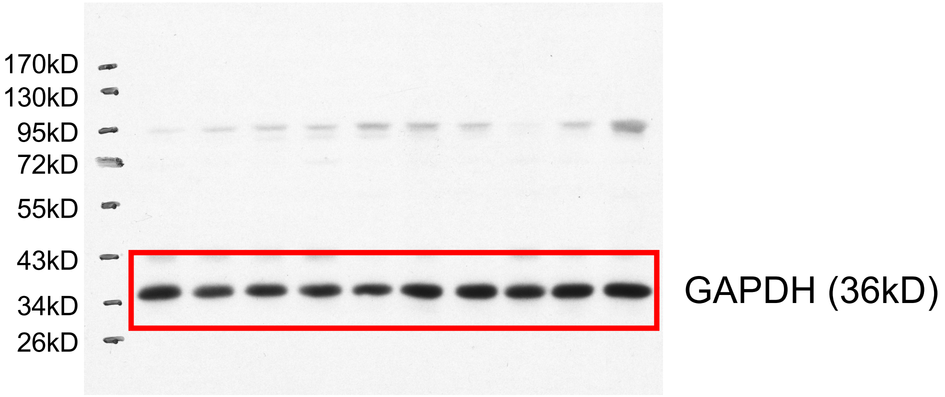

Legend:

Areas of the gel shown in Figure 2C are marked in red.

The membrane was probed first with anti-STAT3 and subsequently with anti-GAPDH antibodies. Predicted molecular weights for STAT3 and GAPDH are indicated.

Full length gels for Figure 4A (part 1)

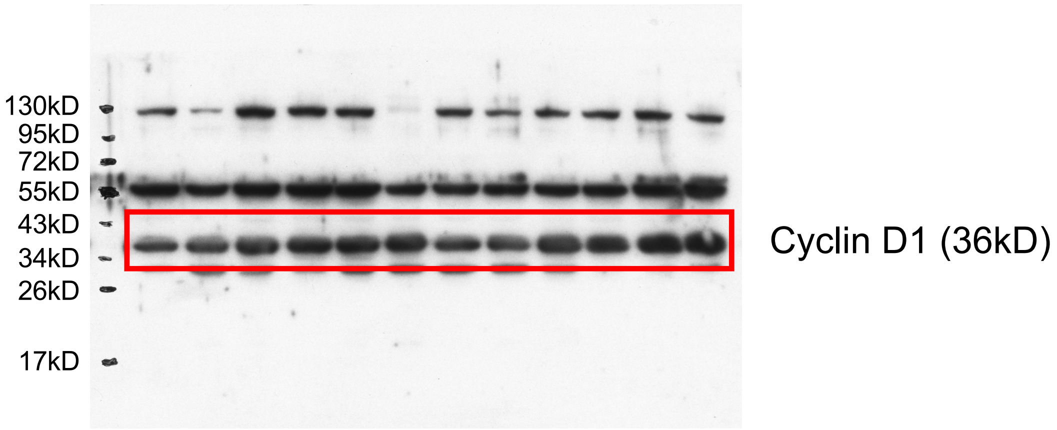

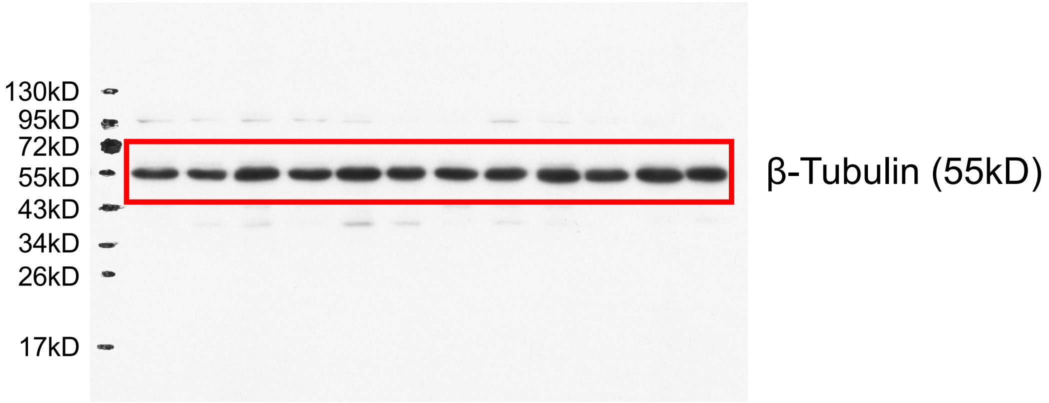

Legend:

Areas of the gel shown in Figure 4A are marked in red.

The membrane was probed first with anti-cyclin D1 and subsequently with anti-Tubulin antibodies. Predicted molecular weights for cyclin D1 and β-tubulin are indicated.

Full length gels for Figure 4A (part 2)

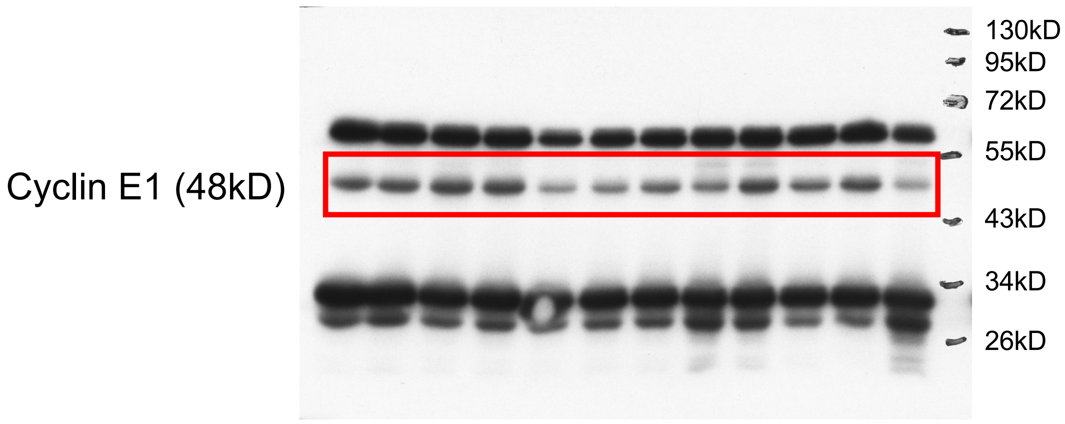

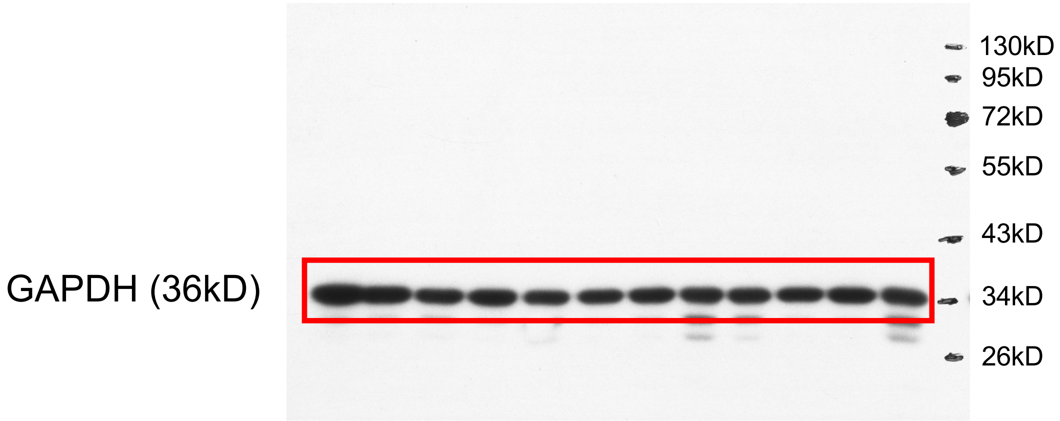

Legend:

Areas of the gel shown in Figure 4A are marked in red.

The membrane was probed first with anti-GAPDH and subsequently with anti-cyclin E1 antibodies. Predicted molecular weights for cyclin E1 and GAPDH are indicated.

Full length gels for Figure 4A (part 3)

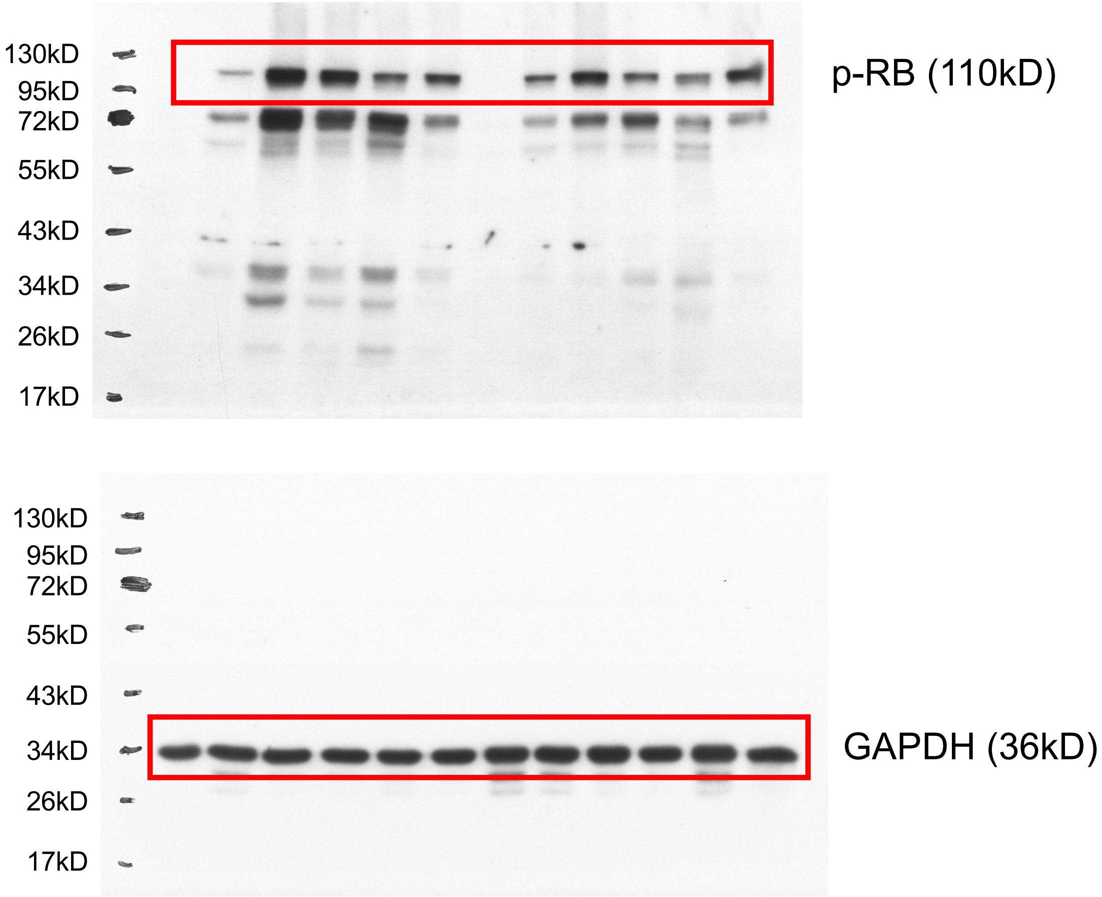

Legend:

Areas of the gel shown in Figure 4A are marked in red.

The membrane was probed first with anti-p-RB and subsequently with GAPDH antibodies. Predicted molecular weights for p-RB and GAPDH are indicated.

Full length gels for Figure 4A (part 4)

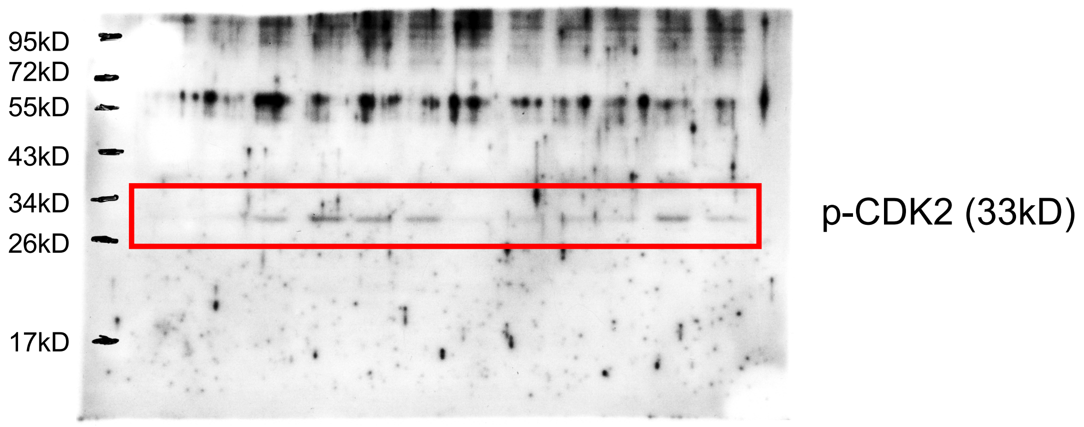

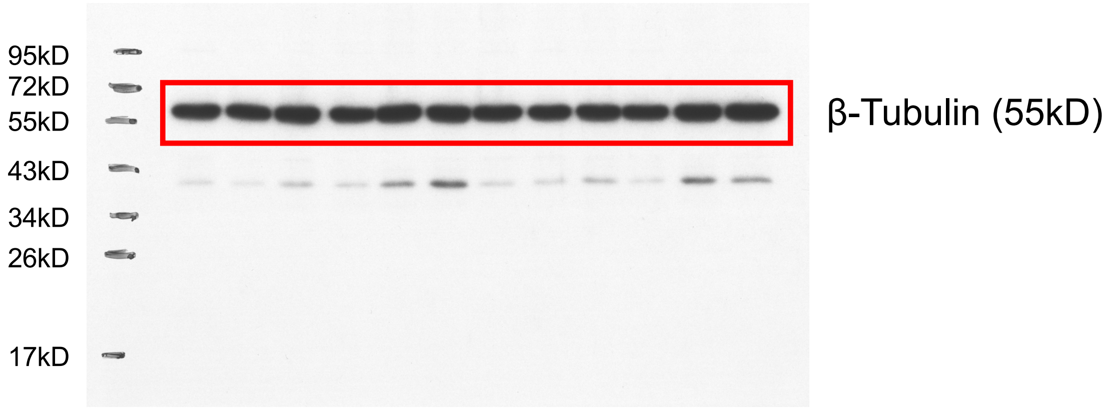

Legend:

Areas of the gel shown in Figure 4A are marked in red.

The membrane was probed first with anti-p-CDK2 and subsequently with anti-Tubulin antibodies. Predicted molecular weights for p-CDK2 and β-tubulin are indicated.

Full length gels for Figure 4A (part 5)

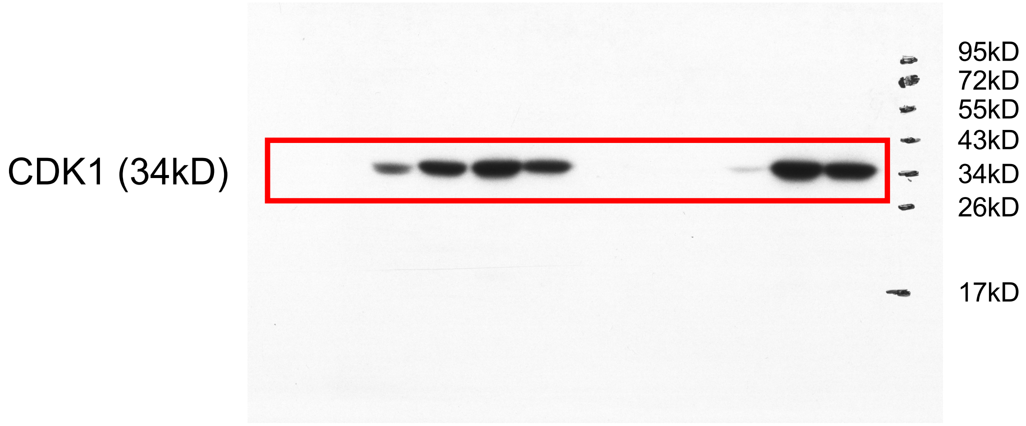

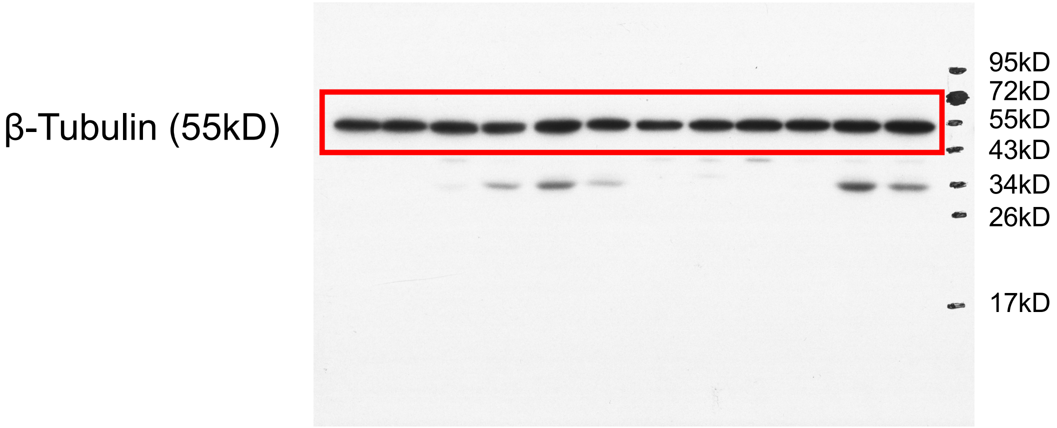

Legend:

Areas of the gel shown in Figure 4A are marked in red.

The membrane was probed first with anti-CDK1 and subsequently with anti-Tubulin antibodies. Predicted molecular weights for CDK1 and β-tubulin are indicated.
